# Insomnia symptoms combined with nocturnal hypoxia associate with cardiovascular comorbidity in the European sleep apnea cohort (ESADA)

**DOI:** 10.1007/s11325-018-1757-9

**Published:** 2018-11-22

**Authors:** Ulla Anttalainen, L. Grote, I. Fietze, R. L. Riha, S. Ryan, R. Staats, J. Hedner, T. Saaresranta, on behalf of the ESADA Study Collaborators, U. Anttalainen, F. Barbé, M. R. Bonsignore, O. Basoglu, P. Bielicki, I. Bouloukaki, Z. Dogas, Z. Dorkova, P. Escourrou, I. Fietze, C. Esquinas, L. Grote, L. Hayes, J. Hedner, P. Joppa, S. Kurki, J. A. Kvamme, R. Tamisier, C. Lombardi, O. Marrone, W. T. McNicholas, J. M. Montserrat, G. Parati, A. Pataka, T. Penzel, J. L. Pépin, M. Pretl, R. L. Riha, G. Roisman, S. Ryan, T. Saaresranta, S. E. Schiza, R. Schulz, P. Sliwinski, R. Staats, M. S. Tasbakan, R. Tkacova, G. Varoneckas, J. Verbraecken, H. Vrints

**Affiliations:** 1 Division of Medicine, Department of Pulmonary Diseases, Turku University Hospital, P.O. Box 52, SF-20521 Turku, Finland; 2 Sleep Research Centre, Department of Pulmonary Diseases and Clinical Allergology, University of Turku, Turku, Finland; 3 Department of Sleep Medicine, Sahlgrenska University Hospital, Gothenburg, Sweden; 4 Center of Sleep Medicine, Charité – Universitätsmedizin Berlin, Luisenstrasse 13, 101 17 Berlin, Germany; 5 Department of Sleep Medicine, Royal Infirmary Edinburgh, 51 Little France Crescent EH, Edinburgh, 164 SA Scotland; 6 Department of Respiratory Medicine, St. Vincent’s University Hospital, Elm Park, Dublin 4, Ireland; 7 Department of Respiratory Medicine, Hospital de Santa Maria, Lisbon, Portugal

**Keywords:** Cardiovascular disease, Comorbidity, Hypoxemia, Insomnia, Sleep apnea, Phenotype

## Abstract

**Purpose:**

The aim of the current study was to further investigate the concept of previously reported high occurrence of comorbidities in obstructive sleep patients (OSA) with insomnia-like symptoms. We hypothesized that this finding at least partly is mediated by nocturnal hypoxia. Moreover, we speculated that the spectrum of the clinical OSA phenotypes differs between European geographical regions.

**Methods:**

Cohort of the European Sleep Apnea Database (*n* = 17,325; 29.9% females) was divided into five subcohorts according to geographical region (North, East, South, West, Central) and further into four clinical presentation phenotypes based on daytime symptoms (EDS) and characteristics suggestive of insomnia.

**Results:**

The insomnia phenotype (alone or together with EDS) dominated in all European regions. Isolated insomnia, however, was less common in the West. Insomnia phenotype was associated with the highest proportion of cardiovascular comorbidity (51.7% in the insomnia vs. 43.9% in the EDS type). Measures of nocturnal hypoxemia were independently associated with cardiovascular comorbidity in phenotypes with insomnia-like symptoms. The burden of comorbidities was high across all geographical regions and clinical phenotypes. Regional differences were clinically relevant for age (48 vs. 54 years), BMI (29 vs. 34 kg/m^2^), and ODI (15 vs. 32/h).

**Conclusion:**

High prevalence of particularly cardiovascular comorbidity among patients with insomnia-like symptoms was linked to nocturnal hypoxemia. Considerable differences in clinical presentation were found among OSA patients across Europe. Our data underline that physicians should ask their patients with suspected OSA also for insomnia symptoms. It remains to be explored if a reduction of nocturnal hypoxemia predicts the improvement of insomnia symptoms.

**Electronic supplementary material:**

The online version of this article (10.1007/s11325-018-1757-9) contains supplementary material, which is available to authorized users.

## Introduction

Obstructive sleep apnea (OSA) is a heterogeneous, complex disorder encompassing a wide variety of symptoms and disorders. A good understanding of the comorbidities and therapeutic outcome in various clinical phenotypes improves the possibility to provide a targeted therapy in each individual patient. We have previously reported on four clinical phenotypes defined by daytime and nighttime symptoms that differ in terms of burden of comorbidity [1]. A phenotype associated with insomnia symptoms was linked to a higher prevalence of cardiovascular diseases (CVD) and other common disorders [1, 2] in a manner that was not explained by the severity of OSA.

The underlying mechanism behind the increased prevalence of cardiovascular comorbidity in the insomnia-like OSA phenotype remains unexplained but some observations have been reported. For instance, patients of the insomnia phenotype were generally older [1]. There are also data suggesting a higher sympathetic activity [3] and association of cardiovascular comorbidity in non-sleepy OSA [4]. Severity of nocturnal hypoxemia could be another explanation. Nocturnal intermittent hypoxia in the current ESADA cohort predicted prevalent hypertension [5] and impaired ventricular relaxation during diastole in another study [6]. Proportion of sleep time spent at an oxygen saturation below 90% was independently associated with an increased risk of hypertension [7] or a higher systolic blood pressure during both sleep and awake in OSA patients [8]. Further, in community-dwelling elderly with OSA, hypoxia was associated with insomnia only in those with CVD [9].

Prevalence estimations of physical and mental disease vary between countries and regions and may impact on comorbidity among OSA phenotypes. For instance, the age-standardized CVD prevalence rates are relatively high in Eastern and Central European countries and lower in Western, Northern, and Southern Europe [10]. Other disorders like chronic depression and insomnia also differ by region [11]. Finally, perception of OSA among health care providers and lay people will determine the characteristics of the patients referred for sleep studies. Therefore, referral patterns among geographical regions are likely to differ and may result in distinct distribution of clinical phenotypes of OSA.

The aim of the current study was to further investigate the concept of different OSA phenotypes. We hypothesized that the previously reported high occurrence of comorbidities in OSA patients with an insomnia phenotype at least in part is mediated by nocturnal hypoxia. Moreover, we speculated that the spectrum of the clinical OSA phenotypes differs between European geographical regions.

## Methods

**Fig. 1:**
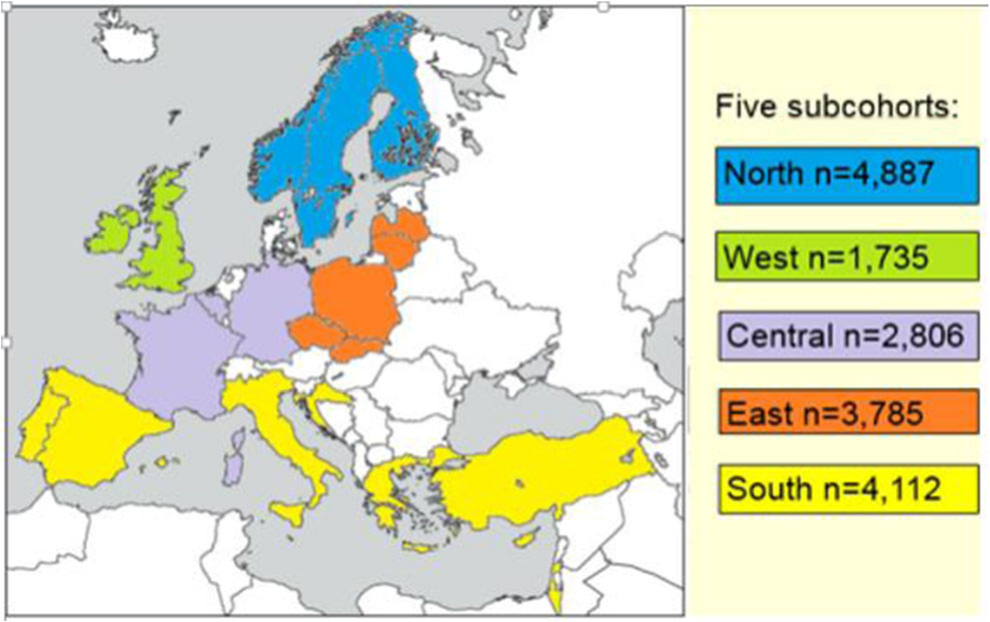
Subcohorts applied in the current study defined by geographical region. *North:* Finland, Norway, Sweden, *East:* Czech Republic, Latvia, Lithuania, Poland, Slovakia, *South:* Greece, Croatia, Israel, Italy, Portugal, Spain, Turkey, *West:* Ireland, UK, *Central:* Belgium, France, Germany

The European Sleep Apnea Database (ESADA) has prospectively collected data from unselected adult patients aged 18 to 80 years referred to several European sleep centers with a history of snoring and other symptoms suggesting OSA like witnessed apneas or increased daytime sleepiness [12]. Comorbidities like cardiovascular, pulmonary, metabolic, and psychiatric diseases based on medical records were also reported to the ESADA database. The study protocol was reviewed and approved by a local ethics committee at each participating center. All patients gave their written, informed consent. Patient data were coded and de-linked before entry into the central database. Data recorded between March 2007 and May 2016 were submitted for analyses. The cohort comprised 19,556 adult patients of which 17,325 (29.9% females) had complete data. The cohort was divided into five subcohorts according to geographical region (North, East, South, West, and Central) (Fig. 1).

The influence of region on clinical patient characteristics (phenotype) was studied after adjustment for age, gender, and BMI. Four clinical phenotypes were defined according to daytime symptoms (EDS) and characteristics suggestive of insomnia (self-reported sleep duration, sleep latency, diagnosed insomnia, or hypnotic use defined by ATC code N05) as reported previously [1]. ATC code N05 includes antipsychotics, anxiolytics, hypnotics, and sedatives. In brief, the criteria for the subgroups were as follows: (1) EDS (daytime+/nighttime−), (2) EDS/insomnia (daytime+/nighttime+), (3) non-EDS/non-insomnia (daytime−/nighttime−), and (4) insomnia (daytime−/nighttime+). Daytime+ indicates that the patient had daytime sleepiness defined by ESS score > 10 and daytime− that ESS score was ≤ 10. Criteria for nighttime+ included at least one of the following: diagnosis of insomnia, self-reported sleep latency ≥ 30 min, self-reported sleep duration ≤ 6 h, or use of hypnotics defined by ATC code N05. Nighttime− referred to situation where none of the nighttime+ criteria were fulfilled. The scoring methods used for polysomnography (PSG) or level 3 cardiorespiratory polygraphy (PG) have been reported previously [1, 12, 13]. A survey was made among participating centers to evaluate possible differences in standard operating procedures (SOPS) of handling referrals or other factors influencing referral patterns. The survey included four questions: possible mandatory screening of sleepiness before referral, categorical reasons for denying CPAP treatment or referrals to sleep studies and finally, which issues may impact on referral patterns for a sleep study.

### Statistical methods

Data are presented as mean ± standard deviation or as frequency (%). Comparisons among the groups or phenotypes were performed using ANOVA for continuous variables, or the chi-square tests for categorical variables. Impact of age, gender, body mass index (BMI), current smoking, nocturnal hypoxia (mean oxygen saturation (SaO_2_)), minimum SaO_2_, oxygen desaturation index (ODI of 4%), apnea hypopnea index (AHI), and geographical region on prevalence of cardiovascular diseases among clinical presentation phenotypes was analyzed by logistic regression. Statistical analyses were performed using IBM SPSS Statistics 22.0 (Armonk, NY, USA: IBM Corp.). *P* value < 0.05 was considered statistically significant. All tests were two-sided.

## Results

### Anthropometric measures, comorbidities, and sleep apnea activity in relation to region and clinical phenotypes

**Fig. 2:**
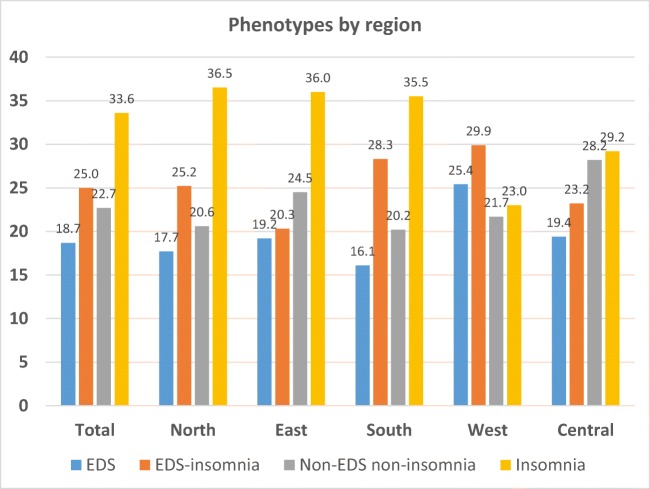
The proportions of four clinical phenotypes of sleep apnea by European regions. *P* value (chi-square test) for trend < 0.001 for all clinical sleep apnea phenotypes

Regional differences were clinically relevant for age (minimum 48 vs. maximum 54 years), BMI (29 vs. 34 kg/m^2^), and ODI (15 vs. 32/h) (Table 1, Online Resource 1). The highest proportion of females was reported in the North region. The youngest, but most obese and sleepy patients, were found in the West, the most severe OSA in the South and the mildest degree of the disorder in the North region. In addition, the prevalence of the four defined clinical phenotypes varied between the European regions (Table 2, Fig. 2). The insomnia phenotype (alone or together with EDS) was the dominant phenotype in all regions. Isolated insomnia, however, was less common in the West.

**Table 1:** Basic characteristics of patients by region

Characteristic	Total	Region					*P* value
		North	East	South	West	Central	
Female gender (%)	29.9	35.6	26.2	28.5	30.3	26.9	< 0.001
Age (years)	52.2 ± 12.6	51.3 ± 13.2	53.6 ± 11.8	53.1 ± 12.5	48.4 ± 12.0	52.9 ± 12.4	< 0.001
BMI (kg/m^2^)	31.3 ± 6.6	29.8 ± 6.0	32.0 ± 6.2	32.4 ± 6.9	34.2 ± 7.8	29.9 ± 5.9	< 0.001
Current smoker (%)	24.2	21.7	23.3	26.9	26.7	24.3	< 0.001
AHI/h	27.0 ± 25.5	15.6 ± 19.2	31.1. ± 26.2	36.0 ± 27.3	23.4 ± 24.6	31.2 ± 24.7	< 0.001
ESS	9.8 ± 5.3	9.8 ± 5.0	9.3 ± 5.5	9.9 ± 5.3	11.2 ± 5.6	9.6 ± 5.2	< 0.001
ODI/h	23.5 ± 25.3	14.7 ± 18.6	29.0 ± 26.6	32.2 ± 28.3	19.7 ± 24.4	20.2 ± 22.9	< 0.001
Mean SaO_2_ (%)	93.1 ± 3.4	93.8 ± 2.4	92.2 ± 4.3	92.6 ± 4.2	93.7 ± 2.1	93.4 ± 2.6	< 0.001
Min SaO_2_ (%)	80.6 ± 10.0	83.3 ± 7.6	78.1 ± 11.3	78.8 ± 11.3	81.6 ± 8.9	80.5 ± 9.2	< 0.001
CVD (%)	48.5	41.6	59.4	51.0	34.6	51.2	< 0.001
Metabolic (%)	34.6	26.5	31.6	42.9	24.6	46.7	< 0.001
Pulmonary (%)	23.4	26.4	25.8	13.8	42.9	28.8	< 0.001
Psychiatric (%)	11.4	11.8	10.5	11.1	14.2	10.9	0.001

*P* value across regions defined by ANOVA except for current smoker, female gender, and proportion of comorbidities with chi-square test. *AHI*, apnea-hypopnea index; *BMI*, body mass index; *CVD*, cardiovascular disease; *ESS*, Epworth Sleepiness Scale; *ODI*, oxygen desaturation index; *SaO*_*2*_, oxyhemoglobin saturation

**Table 2:** Clinical sleep apnea phenotypes by region

Characteristic	Total	Region					*P* value
		North	East	South	West	Central	
EDS (%)	18.7	17.8	19.1	16.1	25.4	19.4	< 0.001
EDS-insomnia (%)	25.0	25.2	20.3	28.3	29.9	23.2	< 0.001
Non-EDS non-insomnia (%)	22.7	20.6	24.5	20.2	21.7	28.2	< 0.001
Insomnia (%)	33.6	36.5	36.0	35.5	23.0	29.2	< 0.001
Combined insomnia and EDS-insomnia (%)	58.6	61.7	56.3	63.8	52.9	52.4	< 0.001

*P* value across regions defined with chi-square test. *EDS*, excessive daytime sleepiness

**Fig. 3:**
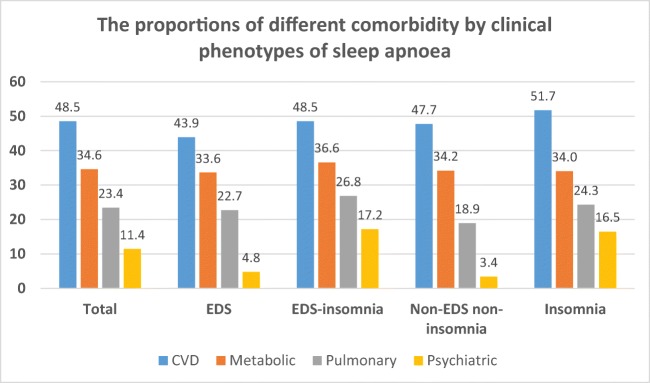
The proportions of cardiovascular, metabolic, pulmonary, and psychiatric comorbidity by four clinical phenotypes of sleep apnea. *P* value (ANOVA) for trend was 0.015 for metabolic disease and < 0.001 for other comorbidities

Clinical phenotypes differed in terms of comorbidity profile. Sleep apnea appeared to be more severe in the EDS group but less severe among those characterized with insomnia (*P* < 0.001). Conversely, cardiovascular morbidity was most prevalent among those with insomnia. A metabolic condition and/or a pulmonary disorder was more common in those with EDS combined with insomnia while the highest prevalence of a psychiatric disorder was found in those with insomnia or insomnia with EDS (Table 3, Fig. 3, Online Resource 2). Insomnia and EDS-insomnia phenotypes were more prevalent among women than men (Table 4).

**Table 3:** Basic characteristics by clinical phenotype

Characteristic		Clinical phenotype				*P* value
	Total	EDS	EDS-ins	Non-EDS non-insomnia	Insomnia	
Female gender (%)	29.9	26.0	32.6	21.3	36.0	< 0.001
Age (years)	52.2 ± 12.6	50.4 ± 12.5	51.6 ± 12.1	52.0 ± 12.8	53.8 ± 12.7	< 0.001
BMI (kg/m^2^)	31.3 ± 6.6	31.8 ± 6.6	32.4 ± 7.0	30.7 ± 6.1	30.8 ± 6.5	< 0.001
Current smoker (%)	24.2	24.3	26.2	22.0	24.1	< 0.001
ESS	9.8 ± 5.3	14.8 ± 3.2	14.8 ± 3.1	6.1 ± 2.7	5.8 ± 2.9	< 0.001
AHI/h	27.0 ± 25.5	31.2 ± 27.8	30.1 ± 27.6	26.4 ± 24.3	22.9 ± 22.7	< 0.001
ODI/h	23.5 ± 25.3	27.7 ± 28.0	27.3 ± 27.6	21.6 ± 23.8	19.5 ± 22.0	< 0.001
Mean SaO_2_ (%)	93.1 ± 3.4	92.7 ± 4.0	92.8 ± 3.6	93.4 ± 3.1	93.4 ± 3.0	< 0.001
Min SaO_2_ (%)	80.6 ± 10.0	78.9 ± 11.3	79.7 ± 10.4	81.2 ± 9.4	81.8 ± 8.9	< 0.001
CVD (%)	48.5	43.9	48.5	47.7	51.7	< 0.001
Metabolic (%)	34.6	33.6	36.6	34.2	34.0	0.015
Pulmonary (%)	23.4	22.7	26.8	18.9	24.3	< 0.001
Psychiatric (%)	11.4	4.8	17.2	3.4	16.5	< 0.001

*P* value defined by ANOVA except for current smoker and female gender and proportion of comorbidities with chi-square test. *AHI*, apnea-hypopnea index; *BMI*, body mass index; *CVD*, cardiovascular disease; *EDS*, excessive daytime sleepiness; *ESS*, Epworth Sleepiness Scale; *ODI*, oxygen desaturation index; *SaO*_*2*_, oxyhemoglobin saturation

**Table 4:** Gender differences in clinical phenotypes

Phenotype	Female, *n* (%)	Male, *n* (%)	Total, *n* (%)	*P* value
EDS	844 (16.2)	2402 (19.7)	3246 (18.7)	< 0.001
EDS-insomnia	1414 (27.2)	2927 (24.1)	4341 (25.0)	< 0.001
Non-EDS non-ins	837 (16.1)	3100 (25.5)	3937 (22.7)	< 0.001
Insomnia	2102 (40.4)	3739 (30.7)	5841 (33.6)	< 0.001

*P* value defined by chi-square test. *EDS*, excessive daytime sleepiness

SOPS of handling referrals did not explain regional differences in clinical phenotypes. Seven sites (two in the South, two in the East, two in the Central, and one in the West region) required screening of sleepiness prior sleep study. None of the sites denied CPAP treatment categorically for patients with specific comorbidities. Knowledge of referring physicians, long waiting lists, and public awareness campaigns regarding sleep in media were reported to influence referral patterns in all regions.

### Predictors of cardiovascular disease in different phenotypes

When measures of nocturnal hypoxemia, age, gender, BMI, smoking, and geographical region were entered in a logistic regression analysis, higher prevalence of CVD was independently associated with lower nadir SaO_2_ in the EDS-insomnia and insomnia phenotypes (Table 5). For example, in EDS-insomnia phenotype, a 1% decrease in nadir SaO_2_ increases the risk of CVD with 1.6%. Accordingly, a 5% lower nadir saturation increases the risk for comorbid CVD by 10.5% ((1.6%)^5). When adding AHI instead of ODI in the logistic regression model, the results remained unchanged (data not shown). Unfortunately, the data on time spent below oxygen saturation of 90% (*T* < 90%) was only available in a subgroup of 7742 patients (44.6% of total). In this subgroup, *T* < 90% explained significantly CVD in univariate analysis in all phenotypes but not after adjustments (data not shown). Additional independent risk factors for CVD included age and BMI among all phenotypes, male gender in all groups except the non-EDS non-insomnia type, and current smoking in the EDS phenotype (Table 5). In terms of geographical regions, the East region had the highest risk for CVD across all phenotypes whereas the West was associated with less prevalent CVD.

**Table 5:** Risk for CVD among clinical phenotypes when adjusted for measures of nocturnal hypoxemia, age, gender, BMI, smoking, and geographical region

Phenotype		EDS		EDS-insomnia		Non-EDS non-insomnia		Insomnia
Adjusted for	HR	95% CI (*P* value)	HR	95% CI (*P* value)	HR	95% CI (*P* value)	HR	95% CI (*P* value)
Mean SaO_2_	1.014	0.983–1.045 (0.390)	1.015	0.984–1.047 (0.348)	0.987	0.950–1.024 (0.481)	1.001	0.970–1.033 (0.966)
Nadir SaO_2_	0.999	0.987–1.011 (0.840)	*0.984*	*0.974–0.995 (0.005)*	0.994	0.982–1.007 (0.378)	*0.988*	*0.978–0.999 (0.035)*
ODI	1.001	0.997–1.006 (0.547)	1.001	0.997–1.005 (0.485)	1.001	0.996–1.006 (0.698)	1.000	0.996–1.004 (0.924)
Age	*1.083*	*1.074–1.092 (< 0.001)*	*1.080*	*1.072–1.088 (< 0.001)*	*1.087*	*1.079–1.095 (< 0.001)*	*1.088*	*1.081–1.095 (< 0.001)*
Male gender	*1.323*	*1.089–1.608 (0.005)*	*1.309*	*1.115–1.536 (0.001)*	1.201	0.995–1.450 (0.056)	*1.402*	*1.221–1.610 (< 0.001)*
BMI	*1.084*	*1.066–1.102 (< 0.001)*	*1.074*	*1.060–1.088 (< 0.001)*	*1.088*	*1.071–1.105 (< 0.001)*	*1.096*	*1.082–1.109 (< 0.001)*
Smoking	*1.279*	*1.046–1.563 (0.016)*	1.005	0.848–1.192 (0.952)	1.078	0.892–1.304 (0.435)	1.158	0.994–1.349 (0.060)
West	1.000		1.000		1.000		1.000	
North	*1.543*	*1.155–2.073 (0.003)*	*1.381*	*1.075–1.774 (0.011)*	*1.733*	*1.283–2.342 (< 0.001)*	*1.862*	*1.417–12.448 (< 0.001)*
East	*2.675*	*1.982–3.611 (< 0.001)*	*2.247*	*1.698–2.972 (< 0.001)*	*3.052*	*2.241–4.156 (< 0.001)*	*2.936*	*2.195–3.927 (< 0.001)*
South	*1.811*	*1.341–2.447 (< 0.001)*	*1.555*	*1.205–2.006 (0.001)*	*1.452*	*1.065–1.981 (< 0.018)*	*2.120*	*1.601–2.808 (< 0.001)*
Central	*1.972*	*1.341–2.447 (< 0.001)*	*1.963*	*1.466–2.629 (< 0.001)*	*1.782*	*1.2941.981 (< 0.001)*	*2.571*	*1.884–3.508 (< 0.001)*

*BMI*, body mass index; *CVD*, cardiovascular disease; *EDS*, excessive daytime sleepiness; *ODI*, oxygen desaturation index; *SaO*_*2*_, oxyhemoglobin saturation

The significant findings are in italic

## Discussion

The present study provides three major findings in patients referred for suspected OSA to European sleep clinics. First, insomnia-like symptoms appeared to dominate the clinical presentation. Second, measures of nocturnal hypoxemia were independently associated with CVD comorbidity in phenotypes with insomnia-like symptoms. Third, single clinical characteristics and clinical OSA phenotypes differed among European geographical regions. However, the burden of comorbidities was high across all geographical regions and clinical phenotypes.

### Association between phenotypes and comorbidities

Although the presence of comorbid insomnia [14, 15] has been recognized in OSA, the endeavor of phenotyping OSA is quite recent [1, 16–18]. The clinical characteristics (ESS score, subjective sleep duration and sleep latency, physician-diagnosed sleep disorder, use of hypnotics) that were applied to phenotype patients in the present study are readily available to clinicians treating patients with OSA. Insomnia-like phenotypes (EDS-insomnia and insomnia) were identified in more than 50% of patients, thereby confirming previous findings [1, 19]. Cardiovascular, pulmonary, and psychiatric comorbidities were more prevalent in phenotypes with insomnia-like symptoms. These results are in line with the findings in an Icelandic OSA cohort using quite similar phenotyping criteria [18] and our previous report [1].

Traditional risk factors such as age, gender, BMI, or smoking explained partly the comorbidity burden among distinct clinical phenotypes in our study. It has been suggested that an increased time lag from the start of the disease to final diagnosis and treatment in these OSA patients with rather atypical symptoms allowed for a higher exposure to harmful OSA-induced cardiovascular consequences [18]. Unfortunately, our database does not allow an assessment of the duration from the first OSA symptoms to diagnosis.

The higher prevalence of cardiovascular comorbidity in phenotypes with insomnia-like symptoms could also be explained by elevated adrenergically mediated alertness manifested as long sleep latency or short self-reported sleep duration. This “hyperarousal”-hypothesis is supported by observations of higher sympathetic activity in non-sleepy patients with severe OSA compared with the sleepy ones [3] as well as in primary insomniacs compared with good sleepers [20]. In fact, previous data has linked cardiovascular comorbidity to non-sleepy OSA in patients with peripheral arterial disease [4]. Epidemiological studies have also confirmed high cardiovascular comorbidity in depressive disorders [21], a condition with frequent symptoms of insomnia. Our data are also in concordance with the literature suggesting that the EDS plus insomnia group is more obese. Insomnia is a potential factor behind weight gain, an observation that has been explained by altered leptin and ghrelin activity and higher glucose and insulin levels as well as an increased appetite [22].

An important novel finding of our study was that nocturnal hypoxia, particularly nadir SaO_2_, was independently associated with higher prevalence of CVD in both phenotypes with insomnia-like symptoms. In the current analysis, three measures of hypoxia were included (ODI, mean, and lowest saturation) in the model and this modifies the statistical power of the single variable AHI or ODI. In fact, when taken as single OSA variable in the analysis, ODI increases the risk for CVD in all phenotype classes. However, it is important to highlight that the nadir of nocturnal hypoxia, a value which is not often in the focus of clinical sleep medicine, was a predictor of CV disease in the insomnia phenotypes. Indeed, recent studies demonstrated that subjects with chronic insomnia have increased sympathetic activity, an impaired sympathetic baroreflex function, and an augmented neural cardiovascular responsiveness to stress [23]. Further, in a Swedish study of community-dwelling elderly subjects with OSA, there was an association between spending more than 1.5% of the sleep time with at a SaO_2_ < 90% and insomnia in those with a CVD [9]. In summary, our data point towards a potential cumulative risk of insomnia and hypoxemia in OSA patients.

### Geographical differences

Our study is the first to address geographical differences in the distribution of the clinical presentation phenotypes in European OSA patients. One of our key findings was that single clinical characteristics and the proportions of distinct clinical presentation phenotypes in the ESADA cohort differed across geographical regions. The finding that insomnia phenotypes are less frequent in Western and Central Europe is in concordance with the report of Dregan and coworkers [24]. In middle-aged and elderly population, they found that insomnia was less common in the western and central part of Europe at least with regard to Ireland and Germany. In the UK, insomnia was similar to Belgium, France, and Bulgaria but higher than in Germany or in northern countries.

The prevalence of comorbidities including cardiovascular disease [25, 26], metabolic syndrome [27], mood disorders [28], and obstructive lung disease [29] was, as expected, high in the ESADA cohort. CVD was most prevalent in the East, metabolic comorbidity in the Central region, and pulmonary and psychiatric comorbidity in the West region, the associations with region and comorbidity being in some cases quite high. The observed differences may at least in part reflect geographical differences in comorbidity according to EU statistics [10, 30, 31]. Also, lifestyle factors such as smoking or physical activity may explain differences [30]. Patients with chronic depression frequently suffer from insomnia and the proportion of the population reporting chronic depression is higher in the northern, western, and central Europe compared to southern and eastern Europe [31]. Particularly high prevalence rates of insomnia have been reported in Poland, Hungary, Estonia, Germany, France, and Portugal but much lower rates in Denmark, Italy, and the Netherlands [11]. In summary, data from this largest cohort or European sleep apnea patients point towards substantial geographical differences in comorbidity and clinical phenotype which may explain the heterogeneity of OSA management between countries. Our data also promote further research to gain deeper insight and to allow better interpretation of the regional effects and risk of comorbidities.

### Referral routines

The management of OSA in Europe is variable [32] and cultural factors, general public, and medical awareness of OSA as well as available diagnostic facilities and treatment options might explain the observed differences. It is also likely that the perception of what constitutes a “typical OSA” patient varies considerably among health care providers in different regions. Although the prevalence rates of EDS and insomnia linked with distinct clinical phenotypes varied considerably, we need to acknowledge that cultural differences in attitudes to sleep may have an impact on how respondents in different countries interpret sleep problems and subsequently rate their sleep [33].

It might be argued that the differences seen in the clinical phenotypes between regions result from referral bias. However, thorough investigation of SOPS to handle referrals did not explain regional differences in clinical phenotypes. On the other hand, for example, media campaigns of awareness of sleep apnea were reported to influence referral patterns in all regions. Further population-based epidemiological studies in the different European regions may help to identify the role of referral bias as the underlying cause for the observed differences in our study.

### Strengths and limitations

The ESADA cohort provides a unique opportunity to explore the real-life clinical practice and characteristics of OSA in different parts of Europe. Although, the ESADA does not reflect the individuals in the general population, they are part of a referral bias which in part may reflect the observed regional differences in clinical phenotypes in Europe. The wide age range and notable female representation in the cohort also allow a consideration of age and gender-related issues. The centralized data monitoring and web-based report format ensure uniformity in the reported data sets. In the ESADA protocol, apneas and hypopneas were significant respiratory events and RERA (respiratory effort related arousals) or RDI (respiratory disturbance index) were not scored. However, phenotypes were not defined based on sleep apnea data. Therefore, the conclusions in terms of regional distribution or comorbidity burden related to phenotypes are considered not to be affected by lack of RERA or RDI. The locally used diagnostic routines applied at participating centers provide a specific methodological challenge (for example, lack of comprehensive pulmonary function tests), which may contribute to differences in the reported comorbidities. A major limitation in our study was the broad definition of insomnia, which did not comply with the ICD or DSM criteria and therefore may lead to overestimation of the prevalence of “real” insomnia. Until now there is no special classification of insomnia with regard to severity but there are first ideas to cluster patients by polysomnography [34] or by the history of disease. However, the finding that even symptoms of insomnia in OSA patients are associated with increased comorbidity is of importance. Although the database does not allow for comprehensive analysis of the effects of sociodemographic factors (for example, socio-economic status, degree of physical exercise, marital status, or caffeine intake) on referral patterns or comorbidity of OSA, it represents the by far most comprehensive description of clinical characteristics in European OSA patients and has revealed a novel finding of link between nocturnal hypoxemia and cardiovascular comorbidity in OSA phenotypes with insomnia-like symptoms. Finally, our finding does not suggest a bias related to SOPs of handling referrals, since the finding was independent of geographical region.

### Clinical implications

Interestingly, comorbid insomnia or insomnia-like symptoms may aggravate the burden of OSA with respect to cognitive function and vigilance. Identifying those patients has implications for personalized treatment. Insomnia-like symptoms have been associated with lower adherence to continuous positive airway pressure (CPAP) therapy [1, 2]. Moreover, treatment effects on outcomes like blood pressure, prevention of cardiovascular events, traffic accident rate, or mood disturbance are likely to differ depending on OSA phenotype. A recent study has demonstrated that cognitive behavioral therapy (CBTi) is effective also in patients with comorbid insomnia and OSA [35]. Combining CBTi with CPAP treatment might improve adherence to CPAP therapy and improve morning restfulness and daytime alertness in patients with OSA [14] and possibly protect from CVD events.

## Conclusions

High prevalence of particularly cardiovascular comorbidity among patients with insomnia-like symptoms was linked to nocturnal hypoxemia. Characteristics of patients referred for suspected OSA differed between European sleep centers independently of confounders like age, gender, and obesity. Considerable differences in clinical presentation were found among OSA patients across Europe. In consideration of the wide generalized spread of patients in the Pan-European database, the ESADA database may turn to be particularly useful for the analysis on how clinical phenotypes may influence treatment outcomes in OSA.

## Electronic supplementary material

ESM 1 (DOCX 20.1 kb)

ESM 2 (DOCX 19.5 kb)
