# Supplementary material for: Insomnia symptoms combined with nocturnal hypoxia associate with cardiovascular comorbidity in the European sleep apnea cohort (ESADA)
Source: Sleep Breath. 2018 Nov 22;23(3):805–14. doi: 10.1007/s11325-018-1757-9 (PMC6700053; doi:10.1007/s11325-018-1757-9)
Supplement: Supplementary file 2 — (DOCX 19.5 kb) [file 11325_2018_1757_MOESM2_ESM.docx]

**Insomnia symptoms combined with nocturnal hypoxia associate with cardiovascular comorbidity in the European sleep apnoea cohort (ESADA)**

Anttalainen U,^1,2^ Grote L,^3^ Fietze I,^4^ Riha RL,^5^ Ryan S,^6^ Staats R^7^, Hedner J,^3^ and Saaresranta T^1,2^ on behalf of the ESADA Study Collaborators

Affiliations of the authors:

1) Division of Medicine, Department of Pulmonary Diseases, Turku University Hospital

2) Sleep Research Centre, Department of Pulmonary Diseases and Clinical Allergology, University of Turku, Turku, Finland

3) Department of Sleep Medicine, Sahlgrenska University Hospital, Gothenburg, Sweden

4) Center of Sleep Medicine**,** Charité – Universitätsmedizin Berlin, Luisenstrasse 13, 101 17 Berlin, Germany

5) Department of Sleep Medicine, Royal Infirmary Edinburgh 51, Little France Crescent EH 164 SA, Scotland

6) Department of Respiratory Medicine, St. Vincent´s University Hospital, Elm Park, Dublin 4, Ireland

7) Department of Respiratory Medicine, Hospital de Santa Maria, Lisbon, Portugal

Address for correspondence:

Ulla Anttalainen

Turku University Hospital

Division of Medicine, Department of Pulmonary Diseases

P.O. Box 52, SF-20521 Turku, Finland

Tel.: +358 50 352 0057

Email: [ulla.anttalainen@tyks.fi](mailto:tasaare@utu.fi)

**Journal: Sleep and Breathing**

**Supplementary Table 2.** Basic characteristics of genders by clinical phenotype.

P-value defined by ANOVA except for current smoker and female gender and proportion of comorbidities with Chi-Square test. AHI=apnoea-hypopnoea index, BMI=body mass index, CVD=cardiovascular disease, EDS=excessive daytime sleepiness, ESS=Epworth Sleepiness Scale, ODI=oxygen desaturation index, SaO_2_= oxyhemoglobin saturation
